# Supplementary material for: Sox9 and Rbpj differentially regulate endothelial to mesenchymal transition and wound scarring in murine endovascular progenitors
Source: Nat Commun. 2021 May 7;12:2564. doi: 10.1038/s41467-021-22717-9 (PMC8105340; doi:10.1038/s41467-021-22717-9)
Supplement: Supplementary file 1 — Supplementary Information [file 41467_2021_22717_MOESM1_ESM.pdf]

Supplementary Information

Sox9 and Rbpj regulate endothelial to mesenchymal transition and scarring in murine endovascular progenitors

Authors: Jilai Zhao[1*], Jatin Patel[1,2*], Simranpreet Kaur[1], Seen-Ling Sim[1], Ho Yi Wong[1], Cassandra Styke[1], Isabella Hogan[1], Sam Kahler[1], Hamish Hamilton[1], Racheal Wadlow[1], James Dight[1], Ghazaleh Hashemi[1], Laura Sormani[1], Edwige Roy[1], Mervin C. Yoder[3], Mathias Francois[4,5], Kiarash Khosrotehrani

Affiliations:

[1]The University of Queensland Diamantina Institute, The University of Queensland, Woolloongabba, QLD 4102, Australia

[2]Centre for Ageing Research Program, Queensland University of Technology, Woolloongabba, QLD 4102, Australia

[3] Indiana Center for Regenerative Medicine and Engineering, Indiana 46202, United States of America

[4] The David Richmond Laboratory for Cardiovascular Development: Gene Regulation and Editing Program, The Centenary Institute, Camperdown, NSW 2050, Australia

[5] The School of Life and Environmental Sciences, Faculty of Science, The University of Sydney, Camperdown, NSW 2006, Australia

a

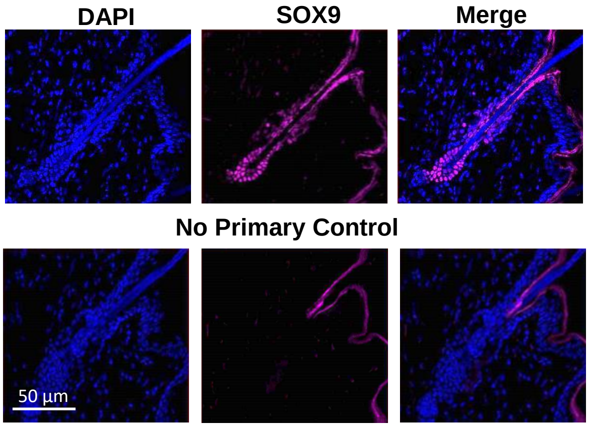
DAPI
SOX9
Merge
No Primary Control
50 µm

b

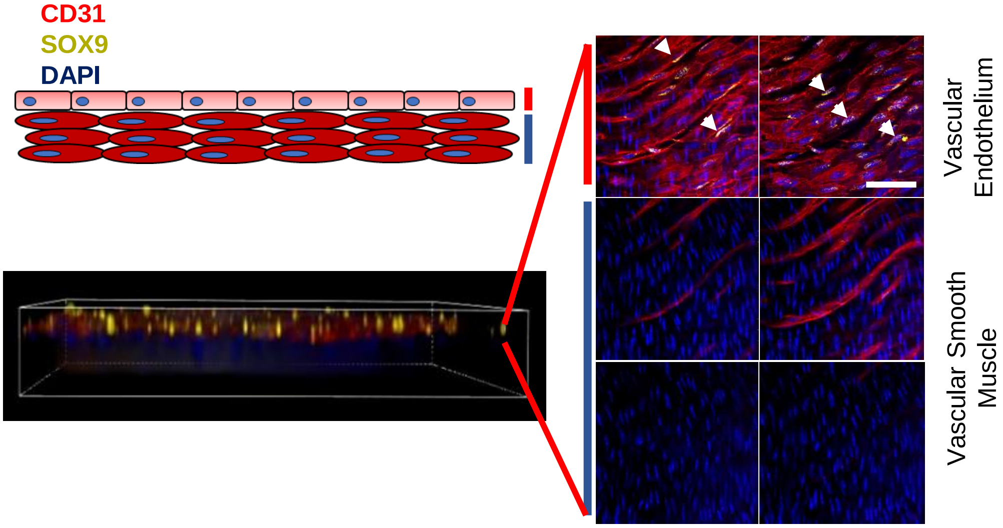
CD31
SOX9
DAPI
Vascular Endothelium
Vascular Smooth Muscle

c

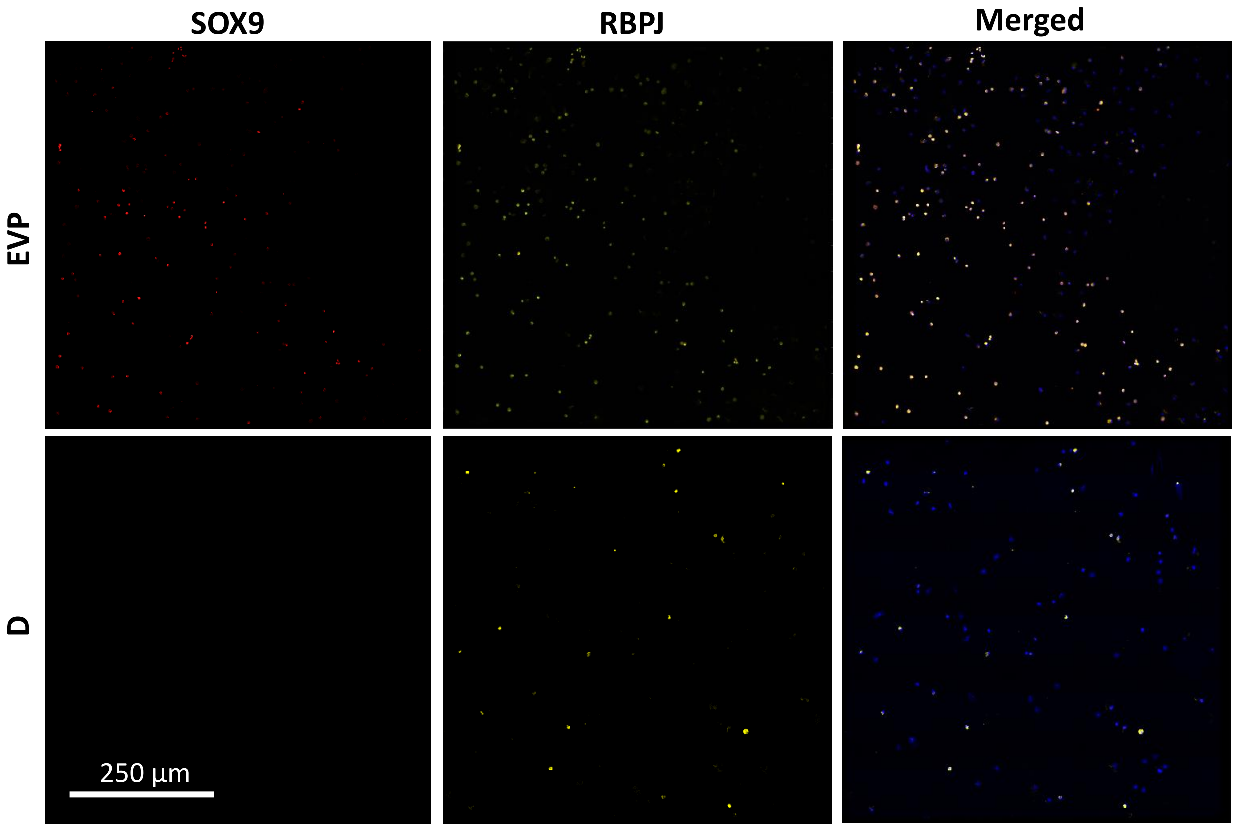
SOX9
RBPJ
Merged
EVP
D
250 µm

Supplementary Figure 1.Specificity of SOX9 antibody and SOX9 expression in the endothelium

a) SOX9 immunostaining in skin, showing positive staining within resting telogen hair-follicles mostly around the bulge area. Scale bar represents 100µm.

b) SOX9 staining in whole mount aorta, demonstrating the positive staining in the endothelial layer co-localising with CD31.

c) Large tile-scan of sorted aortic EVPs and D cells stained for DAPI (blue) SOX9 and RBPJ showing increase SOX9 and RBPJ staining in the EVP population.

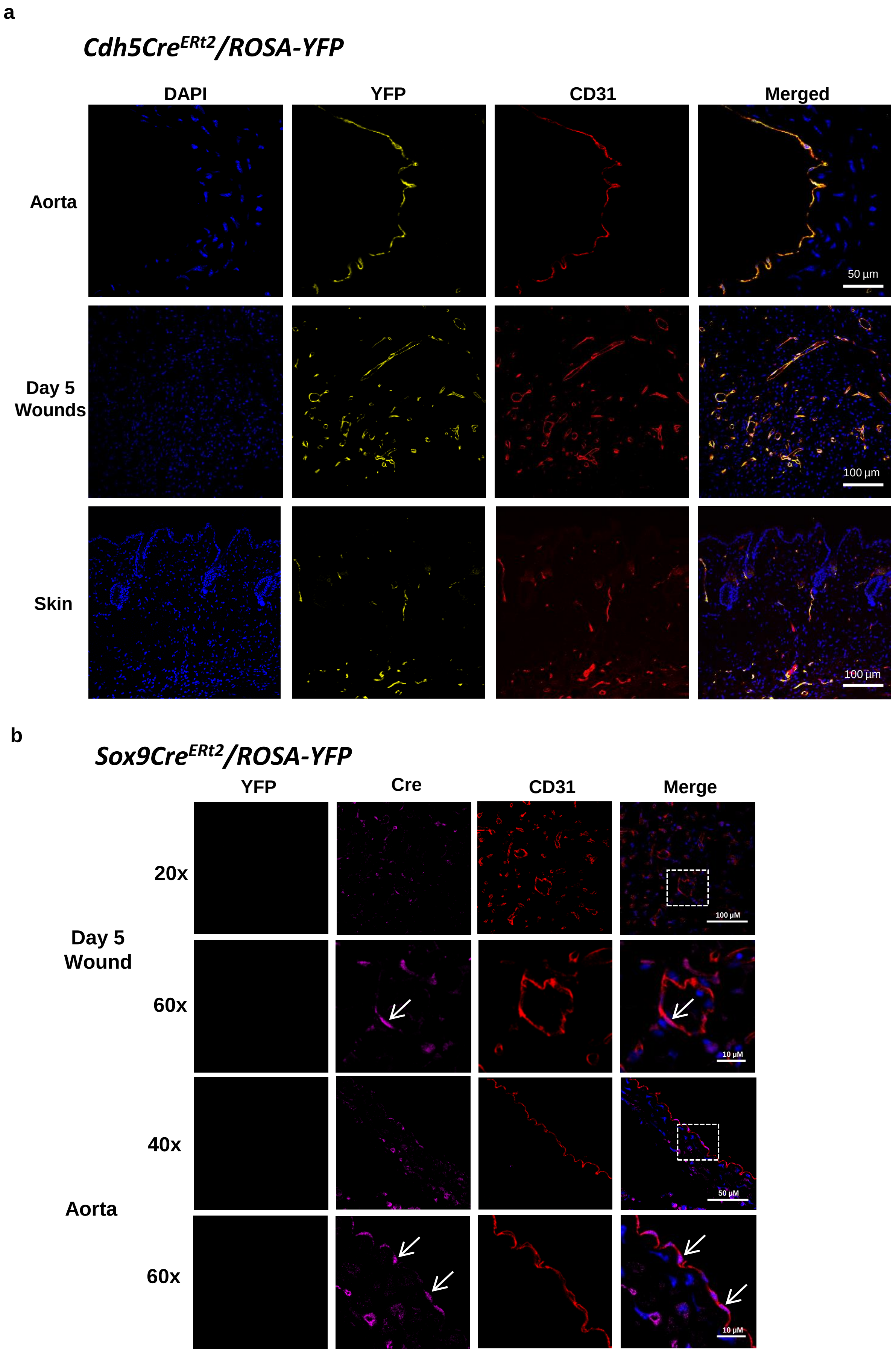
a
Cdh5CreERt2/ROSA-YFP
DAPI
YFP
CD31
Merged
Aorta
Day 5 Wounds
Skin
50 µm
100 µm
100 µm
b
Sox9CreERt2/ROSA-YFP
YFP
Cre
CD31
Merge
Day 5 Wound
20x
60x
Aorta
40x
60x
100 µM
10 µM
50 µM
10 µM

Supplementary Figure 2. Specificity of the Cre/ER model

a) *Cdh5Cre*^{ER}*/RosaYFP* mice were injected with tamoxifen and immediately collected for analysis in homeostasis (skin and aorta) or after wounding (D5 wound): Immunofluorescent images of Cre-activated YFP and pan-endothelial marker CD31 expression within a range of organ beds show colocalization in all scenarios ensuring endothelial staining.

b) Sox9 reporter model further demonstrates Sox9 expression in the endothelium. Immunofluorescent images of nuclear Cre expression driven by the *sox9* promoter within the granulation tissue and aorta of *Sox9Cre*^{ER}*/RosaYFP* mice co-localizing to the vasculature. Of interest, despite tamoxifen delivery, this model did not allow YFP expression.

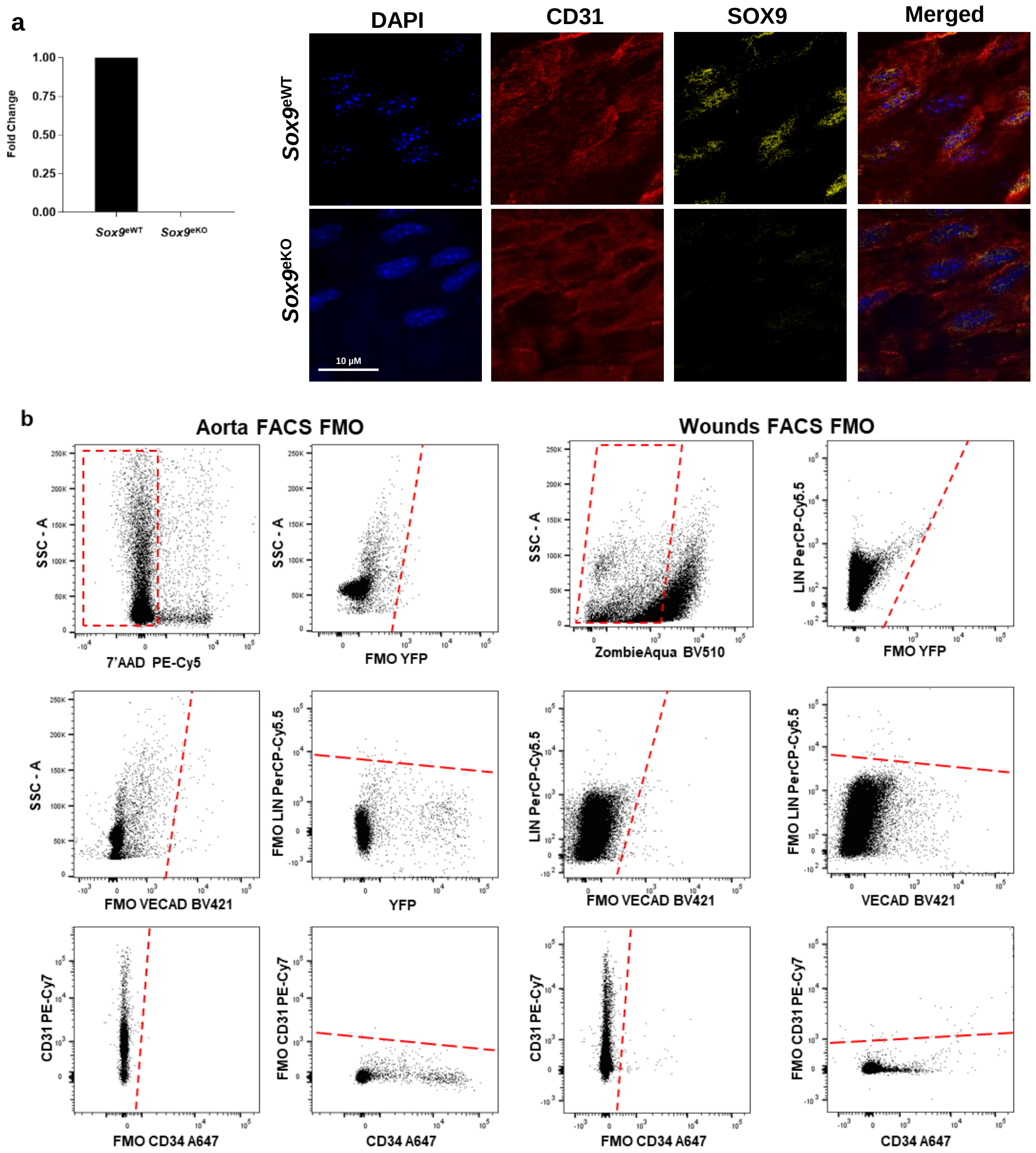

Supplementary Figure 3. Validation of SOX9 knockout and fluorescent-minus-one controls

a) Validation of conditional deletion of Sox9, Loss of SOX9 expression in endothelial cells following conditional knockout using the *sox9*$^{fl/fl}$*.Cdh5Cre*^{ER}*/RosaYFP* (*Sox9*^{eKO}) *mice.*

b) Fluorescence-minus-one (FMO) controls established to conduct flow cytometry analysis in the aorta and wounds.

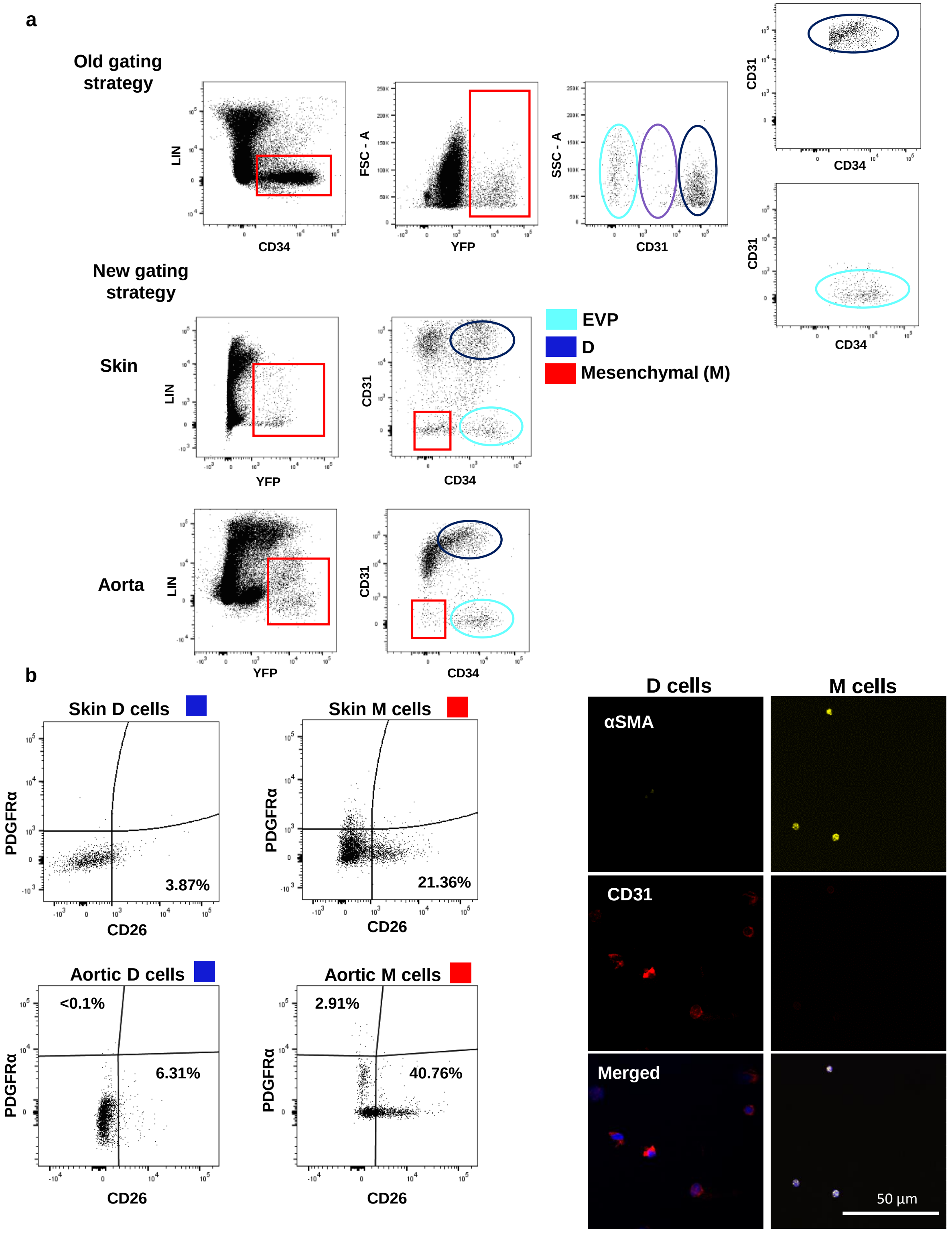

Supplementary Figure 4. Endovascular progenitor FACS gating and validation

a) Flow cytometry strategy of a newly established gating strategy to identify an additional M population within the total endothelial fraction (Lin^{neg}YFP+CD31^{neg}CD34^{neg}).

b) Surface expression of key mesenchymal markers (CD26 and PDGFRα) are at a higher level in the M population compared to differentiated endothelial D cells. Sorted D and M cells shows exclusive expression of αSMA within the M population whereas M cells lack CD31.

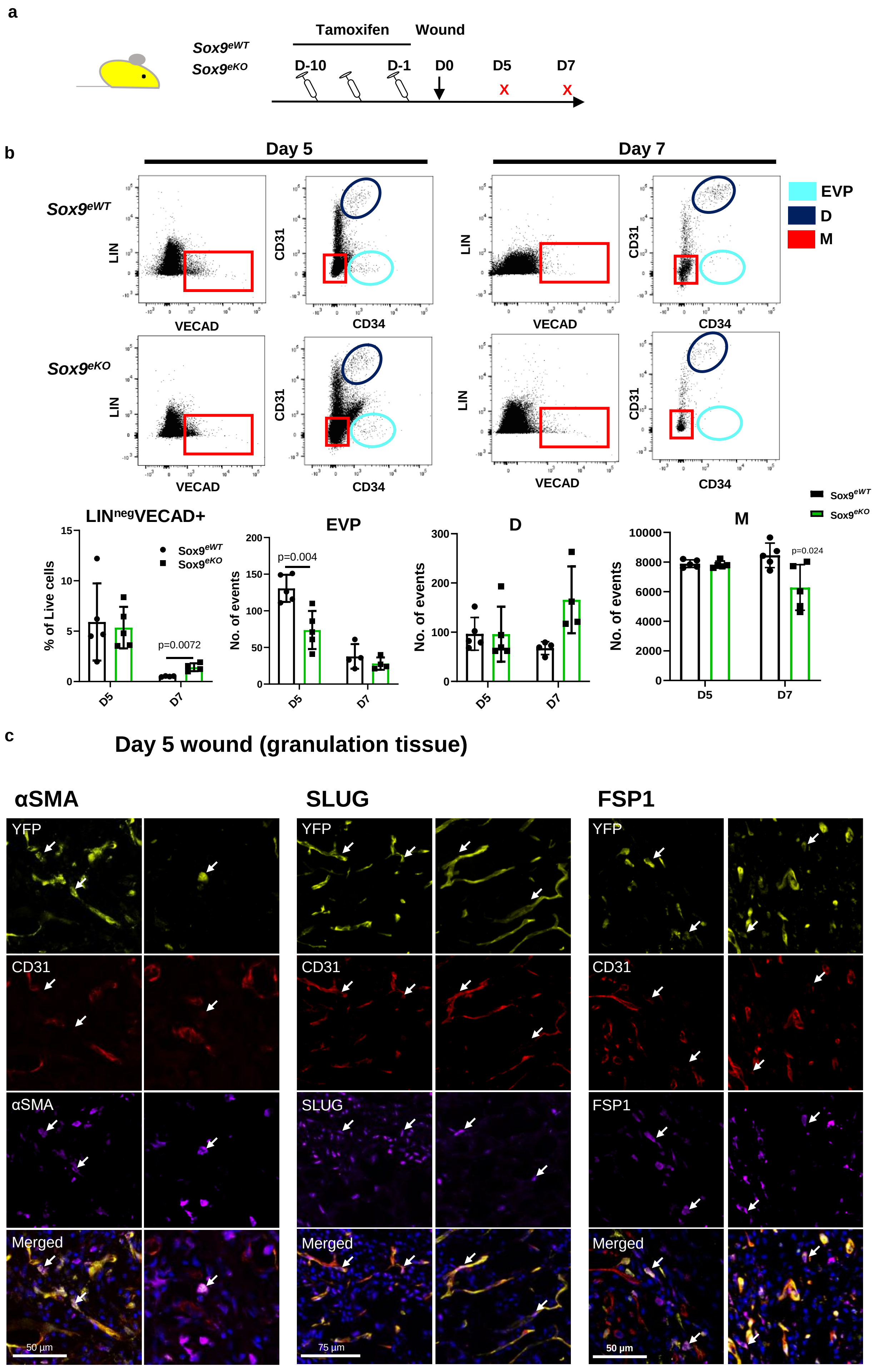
a
Sox9eWT
Sox9eKO
Tamoxifen
Wound
D-10
D-1
D0
D5
D7
b
Day 5
Day 7
Sox9eWT
Sox9eKO
LIN
VECAD
CD31
CD34
EVP
D
M
LINnegVECAD+
% of Live cells
p=0.0072
EVP
No. of events
p=0.004
D
M
p=0.024
D5
D7
c
Day 5 wound (granulation tissue)
αSMA
SLUG
FSP1
YFP
CD31
Merged
50 µm
75 µm

Supplementary Figure 5. EndMT evaluation in *Sox9eKO* wounds over time

a) Schematic representation demonstrating experimental set up using conditional vascular specific *Sox9* knockout mice (*Sox9j*$^{fl/fl}$*/Cdh5-Cre*^{ER} *RosaYFP – Sox9*^{eKO}) in the full-thickness 6mm punch wound model.

b) FACS gating strategy to identify the total endothelial population (Lin^{neg}VECAD+), EVPs, D and M cells at D5 and D7 within the granulation tissue. The number of total endothelial cells significantly decreased over time, however *Sox9*^{eKO} resulted in a larger total endothelial population in the D7 wound compared to *Sox9*^{eWT}. The number of EVPs was also significantly reduced within the *Sox9*^{eKO} D5 wound as well as a decrease in the M population within the D7 wound. (*$p<0.05$ **$p<0.01$ vs Sox9^{eWT}; n=5 biologically independent animals; mean ± SD; p value was calculated by multiple two-tailed unpaired t tests).

c) Immunofluorescence staining of endothelial markers CD31 and YFP with EndMT markers SLUG, and αSMA and FSP1 demonstrates their co-localisation with YFP+ cells within the D5 wound.

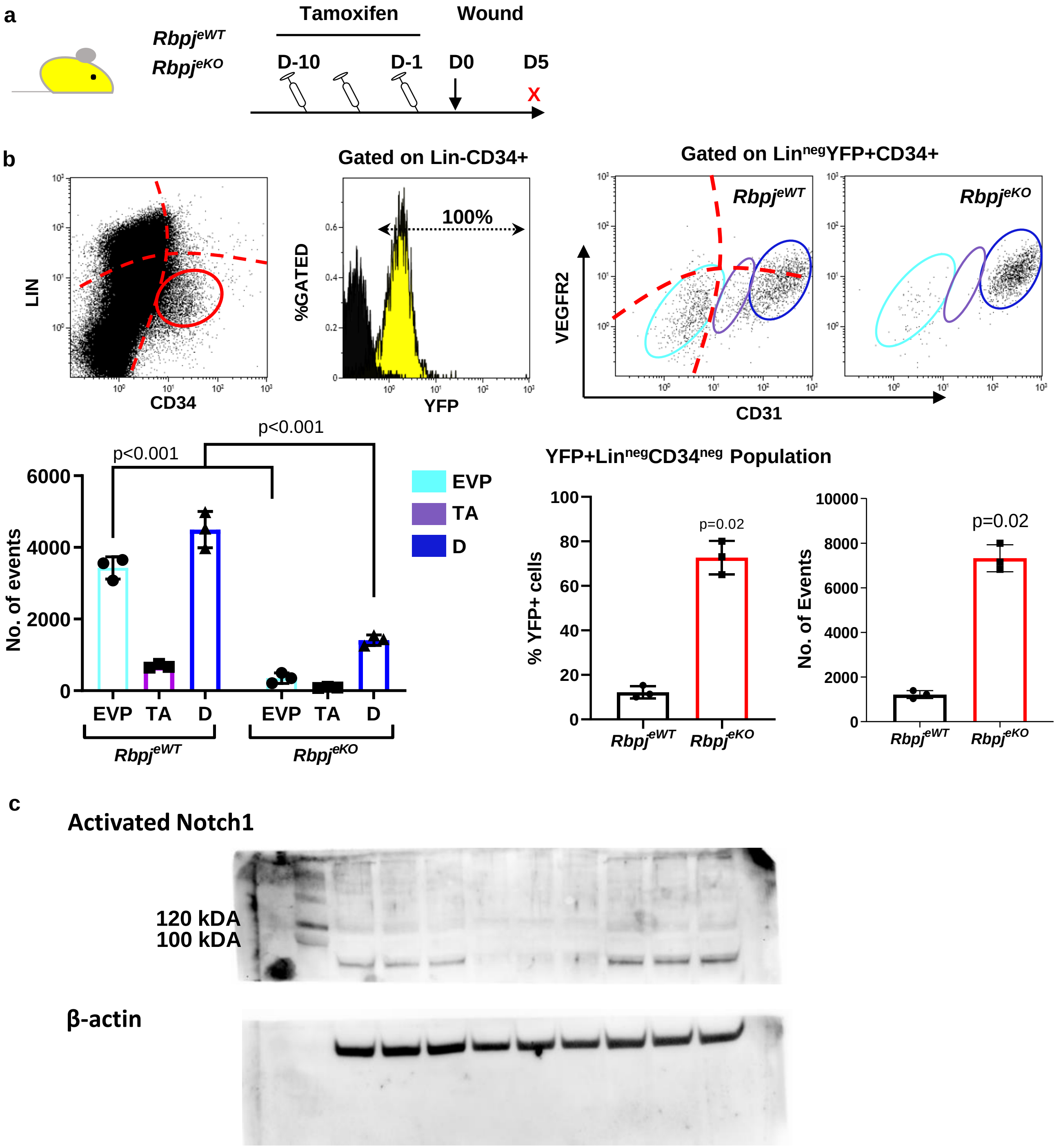

a
Rbpj^eWT
Rbpj^eKO
Tamoxifen
Wound
D-10
D-1
D0
D5
X
b
Gated on Lin-CD34+
Gated on Lin^neg YFP+CD34+
LIN
CD34
%GATED
100%
YFP
VEGFR2
CD31
Rbpj^eWT
Rbpj^eKO
p<0.001
p<0.001
6000
4000
2000
0
No. of events
EVP
TA
D
EVP TA D
Rbpj^eWT
EVP TA D
Rbpj^eKO
YFP+Lin^neg CD34^neg Population
% YFP+ cells
100
80
60
40
20
0
p=0.02
Rbpj^eWT
Rbpj^eKO
No. of Events
10000
8000
6000
4000
2000
0
p=0.02
Rbpj^eWT
Rbpj^eKO
c
Activated Notch1
120 kDA
100 kDA
β-actin

Supplementary Figure 6. EndMT evaluation in *RbpjeKO* wounds

a) Schematic diagram demonstrating experimental set up using conditional vascular specific *Rbpj* knockout mice (*$Rbpj^{fl/fl}$/Cdh5-Cre^{ER} RosaYFP – $Rbpj^{eKO}$*).

b) Flow cytometry plots showing the wound vascular endothelium harbour a distinct CD34 positive, lineage (Lin) negative population (red gate) that is entirely YFP. Three distinct populations were observed based on CD31 and VEGFR2 expression showing the endothelial hierarchy and changes between wild-type controls (*$Rbpj^{eWT}$*) and *$Rbpj^{eKO}$* mice after 10 days tamoxifen induction. ($p<0.001$ vs $Rbpj^{eWT}$; n=3 biologically independent animals; mean ± SD; p value was calculatedby 2-way ANOVA with multiple comparison of row mean). Flow cytometry analysis showed increased loss of endothelial phenotype amongst YFP+ cells from the *$Rbpj^{eKO}$* mice as demarcated by the loss of CD34 expression. ($p=0.02$ vs $Rbpj^{eWT}$; n=3 biologically independent animals; mean ± SD; p value was calculated by two-tailed unpaired t test)

c) Original immunoblot from Fig 5C. Ladder marker sizes shown on the left. Activated Notch1 expected molecular weight is 80 kDA. β-actin expected molecular weight is 42 kDA. Two membranes were derived from one gel and cut and 55 kDA, imaged separately.

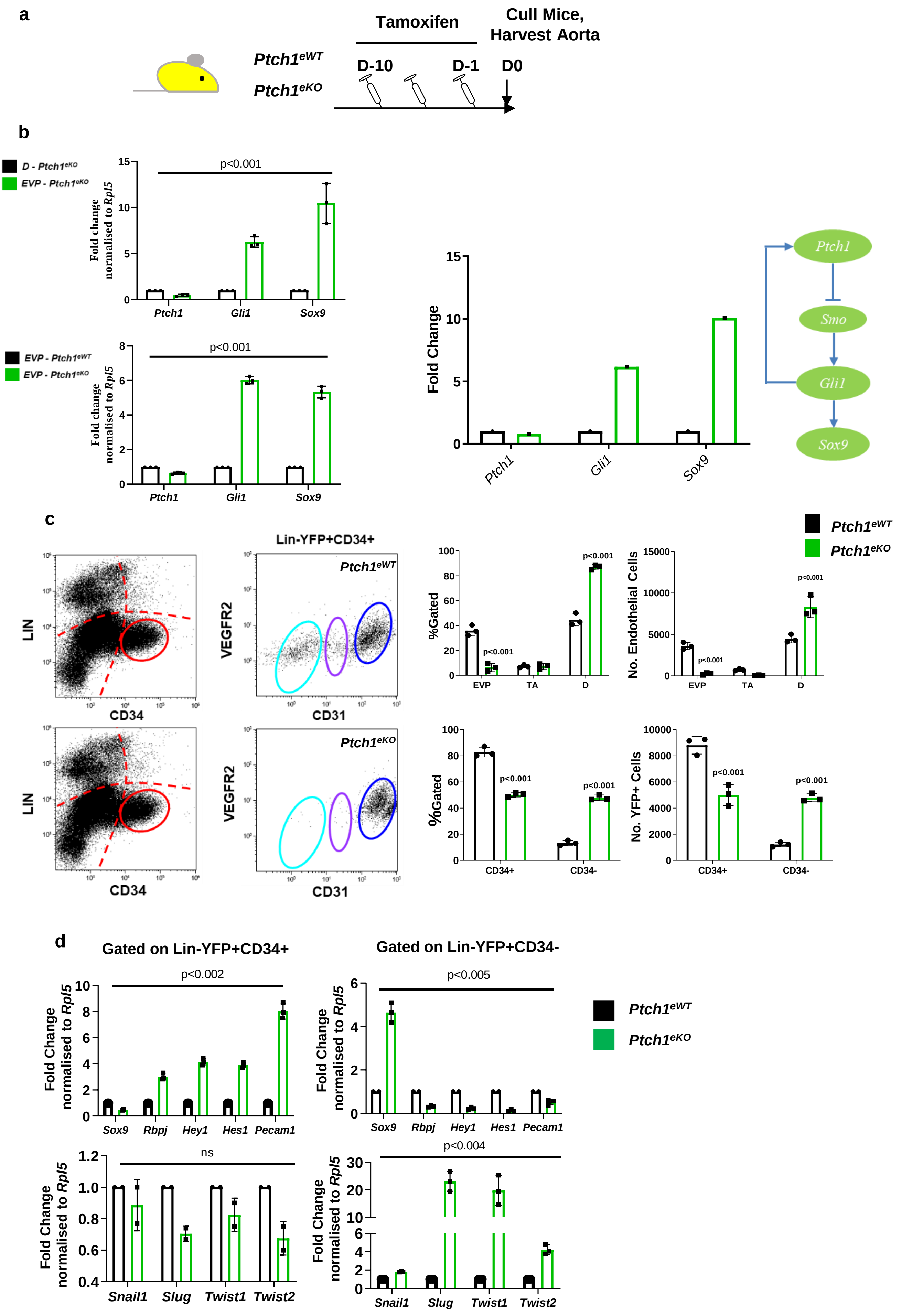
a
Tamoxifen
Cull Mice, Harvest Aorta
Ptch1eWT
Ptch1eKO
D-10
D-1
D0
b
D - Ptch1eKO
EVP - Ptch1eKO
Fold change normalised to Rpl5
p<0.001
Ptch1
Gli1
Sox9
EVP - Ptch1eWT
EVP - Ptch1eKO
Fold Change
Ptch1
Smo
Gli1
Sox9
c
Lin-YFP+CD34+
LIN
CD34
VEGFR2
CD31
%Gated
No. Endothelial Cells
EVP
TA
D
CD34+
CD34-
No. YFP+ Cells
d
Gated on Lin-YFP+CD34+
Gated on Lin-YFP+CD34-
Fold Change normalised to Rpl5
p<0.002
p<0.005
ns
p<0.004
Sox9
Rbpj
Hey1
Hes1
Pecam1
Snail1
Slug
Twist1
Twist2

Supplementary Figure 7. Molecular characterisation of the hedgehog pathway in endovascular progenitors

a) Schematic diagram demonstrating experimental set up using conditional vascular specific *Ptch1* knockout mice $Ptch1^{fl/fl}$/*Cdh5-*Cre^{ER} *RosaYFP* ($Ptch1^{eKO}$).

b) qPCR analysis of key hedgehog-signalling genes in EVP vs D and $Ptch1^{eKO}$ and wild-type controls $Ptch1^{eWT}$ within the aorta. Previously published bulk RNA sequencing of EVPs vs D cells in WT mice shows up-regulated hedgehog signalling constituents in the progenitor fraction (**$p<0.01$; ***$p<0.001$ vs $Ptch1^{eWT}$; n=3; cells were sorted from 3 groups of 5 biologically independent animals mean ± SD; p values were calculated by 2-way ANOVA with multiple comparison of row mean).

c) Flow cytometry plots showing the aortic endothelium harbour a distinct CD34 positive, lineage (Lin) negative population (red gate) that is entirely YFP positive showing the endothelial hierarchy and changes between wild-type controls ($Ptch1^{eWT}$) and $Ptch1^{eKO}$ mice after 10 days tamoxifen. Percentage gated and number of EVP and total CD34 positive cells is significantly reduced in $Ptch1^{eKO}$. A significant increase in CD34 negative is observed in $Ptch1^{eKO}$ ($p<0.001$ vs $Ptch1^{eWT}$; n= 3 biologically independent animals; mean ± SD; p values were calculated by 2-way ANOVA with multiple comparison of row mean).

d) qPCR analysis conducted to compare key endothelial and EndMT genes between Lin-YFP+CD34+ and Lin-YFP+CD34- cells from $Ptch1^{eKO}$ compared to $Ptch1^{eWT}$ (***$p<0.001$ vs $Ptch1^{eWT}$; n=3; cells were sorted from 3 groups of 5 biologically independent animals mean ± SD; p values were calculated by 2-way ANOVA with multiple comparison of row mean).

Supplementary Table 1: siRNA sequence

**siRNA**	***Sox9***	**Scrambled**
Sequence 1	5'- GGGUGAGCUUUGAUUAAUU -3'	5'- UGGUUUACAUGUCGACUAA -3'
Sequence 2	5'- CGUUUAACCUUCAAGAAU -3'	5'- UGGUUUACAUGUUUUCUGA -3'
Sequence 3	5'- GUAUGGUCAUCUGUUGUUA -3'	5'- UGGUUUACAUGUUUUCCUA -3'
Sequence 4	5'- CCUUCGACGUCAAUGAGUU -3'	5'- UGGUUUACAUGUUGUGUGA -3'

Supplementary Table 2: qPCR Primers

Mouse primers

**Target**	**Forward Primer**	**Reverse Primer**
Rpl5	GGCGGCGAGAGGGTAAAA	GCACAGACGATCATATCCCCTTC
Ptch1^{Non}	GGACCGTGTCCTGAGGTGTCT	GGCAAACCGGACGACACTT
Ptch2	CTCCGCACCTCATATCCTAGC	TCCCAGGAAGAGCACTTTGC
Gli1	GCATGGGAACAGAAGGACTTTC	CCTGGGACCCTGACATAAAGTT
Gli2	AGAAGTCTCCATCTCAGAGGCTCA	CACCTGCATGCTAGAGGCAAA
Sox9	AGTACCCGCATCTGCACAAC	ACGAAGGGTCTCTTCTCGCT
Slug	ACTGGACACACACACAGTTATT	ACTTACACGCCCCAAGGATG
Twist1	AGCTGAGCAAGATTCAGACC	CATCTTGGAGTCCAGCTCGT
Twist2	CCAGGTACATAGACTTCCTC	GAGAAGGCGTAGCTGAGACG
Snail1	GCGGAGTTGACTACCGACC	GAAGGTGAACTCCACACACG
Hey1	GCGCGGACGAGAATGGAAA	TCAGGTGATCCACAGTCATCTG
Hes1	CCAGCCAGTGTCAACACGA	AATGCCGGGAGCTATCTTTCT
Il33	ATTTCCCCGGCAAAGTTCAG	AACGGAGTCTCATGCAGTAGA
p16	GCAGGTTCTTGGTCACTGT	TGTTCACGAAAGCCAGAGCG
p21	CCTGGTGATGTCCGACCTG	CCATGAGCGCATCGCAATC
p57	AGAGAACTGCGCAGGAGAAC	TCTGGCCGTTAGCCTCTAAA
Rbpj	AGTTGCACAGAAGTCTTACGG	CCTATTCCAATAAACGCACAGGG
TGFβ	GACCGCAACAACGCCATC	TCTGCACGGGACAGCAATG
Pecam1	GCCAAGGCCAAACAGAAAC	CTTCCACACTAGGCTCAGAAAT

Human primers

**Target**	**Forward Primer**	**Reverse Primer**
HPRT	CCTGGCGTCGTGATTAGTGAT	AGACGTTCAGTCCTGTCCATAA
SOX18	CGCGTGTATGTTTGGTTC	ATGTAACCCTGGCAACTC
SOX9	AGCGAACGCACATCAAGAC	CTGTAGGCGATCTGTTGGGG
IL33	CCACTGAGGAAAGAGCCA	TGAGCCTATCGTTTGGAACTG
HEY1	TGGAGAGGCGCCGCTGTAGTTA	CAAGGGCGTGCGCGTCAAAGTA
HES1	GTCAACACGACACCGGATAA	TTCAGCTGGCTCAGACTTTC
