# Supplementary material for: Sox9 and Rbpj differentially regulate endothelial to mesenchymal transition and wound scarring in murine endovascular progenitors
Source: Nat Commun. 2021 May 7;12:2564. doi: 10.1038/s41467-021-22717-9 (PMC8105340; doi:10.1038/s41467-021-22717-9)
Supplement: Supplementary file 3 — Reporting Summary [file 41467_2021_22717_MOESM3_ESM.pdf]

nature research

Corresponding author(s): Kiarash Khosrotehrani

Last updated by author(s): 17/03/2021

Reporting Summary

Nature Research wishes to improve the reproducibility of the work that we publish. This form provides structure for consistency and transparency in reporting. For further information on Nature Research policies, see our Editorial Policies and the Editorial Policy Checklist.

Statistics

For all statistical analyses, confirm that the following items are present in the figure legend, table legend, main text, or Methods section.

n/a	Confirmed	
☐	☒	The exact sample size (n) for each experimental group/condition, given as a discrete number and unit of measurement
☐	☒	A statement on whether measurements were taken from distinct samples or whether the same sample was measured repeatedly
☐	☒	The statistical test(s) used AND whether they are one- or two-sided<br>*Only common tests should be described solely by name; describe more complex techniques in the Methods section.*
☒	☐	A description of all covariates tested
☐	☒	A description of any assumptions or corrections, such as tests of normality and adjustment for multiple comparisons
☐	☒	A full description of the statistical parameters including central tendency (e.g. means) or other basic estimates (e.g. regression coefficient) AND variation (e.g. standard deviation) or associated estimates of uncertainty (e.g. confidence intervals)
☐	☒	For null hypothesis testing, the test statistic (e.g. F, t, r) with confidence intervals, effect sizes, degrees of freedom and P value noted<br>*Give P values as exact values whenever suitable.*
☒	☐	For Bayesian analysis, information on the choice of priors and Markov chain Monte Carlo settings
☒	☐	For hierarchical and complex designs, identification of the appropriate level for tests and full reporting of outcomes
☒	☐	Estimates of effect sizes (e.g. Cohen's d, Pearson's r), indicating how they were calculated

Our web collection on statistics for biologists contains articles on many of the points above.

Software and code

Policy information about availability of computer code

Data collection	FACSDiva software version 5.0.3, Kaluza version 2.1(flow cytometry)<br>Nikon [NIS Elements imaging software] version 4.50, Flouview FV3000 software v1.0 (microscopic IHC and IF images), OlyVIA v2.9.1 (polarised light)<br>QuantStudio version 7 (RT-qPCR)<br>Biorad ChemiDock Imaging System; ImageLab v6.1, Licor Odyssey Clx Imaging system; Image Studio v5.0(western blot)
Data analysis	FlowJo version v10 (Flow cytometry)<br>ImageJ v1.53h, Nikon [NIS elements] v4.06.00 (IF images)<br>OlyVIA V3.2.1 (polarised images)<br>Visiopharm software v2018.9<br>GraphPad Prism software v8

For manuscripts utilizing custom algorithms or software that are central to the research but not yet described in published literature, software must be made available to editors and reviewers. We strongly encourage code deposition in a community repository (e.g. GitHub). See the Nature Research guidelines for submitting code & software for further information.

Data

Policy information about availability of data

All manuscripts must include a data availability statement. This statement should provide the following information, where applicable:

- Accession codes, unique identifiers, or web links for publicly available datasets
- A list of figures that have associated raw data
- A description of any restrictions on data availability

The data that support the findings of this study are available from the corresponding author upon reasonable request.

Field-specific reporting

Please select the one below that is the best fit for your research. If you are not sure, read the appropriate sections before making your selection.

[x] Life sciences [ ] Behavioural & social sciences [ ] Ecological, evolutionary & environmental sciences

For a reference copy of the document with all sections, see nature.com/documents/nr-reporting-summary-flat.pdf

Life sciences study design

All studies must disclose on these points even when the disclosure is negative.

Sample size	No pre-determination of sample size was taken. However, once statistical significance was observed between experimental groups the experiment was stopped. This is due to transgenic mouse models being used and ensuring minimal numbers as possible was being adopted to reduce over use of animals and maintaining ethical constraints.
Data exclusions	No data were excluded from result and analysis
Replication	The number of relevant biological or experimental replicates are stated in each respective figure legends Results were replicated over different time points and was successful.
Randomization	Samples and specimens were not randomly allocated. Genotype of mouse strains were used to allocate samples into respective groups within an appropriate age range (6 - 8 weeks old) regardless of sex
Blinding	Single blinding of group allocation was conducted for the analysis of immuno-fluorescence, polarised collagen and macroscopic scar size quantification

Reporting for specific materials, systems and methods

We require information from authors about some types of materials, experimental systems and methods used in many studies. Here, indicate whether each material, system or method listed is relevant to your study. If you are not sure if a list item applies to your research, read the appropriate section before selecting a response.

Materials & experimental systems

n/a	Involved in the study	
[ ]	[x]	Antibodies
[ ]	[x]	Eukaryotic cell lines
[x]	[ ]	Palaeontology and archaeology
[ ]	[x]	Animals and other organisms
[ ]	[x]	Human research participants
[x]	[ ]	Clinical data
[x]	[ ]	Dual use research of concern

Methods

n/a	Involved in the study	
[x]	[ ]	ChIP-seq
[ ]	[x]	Flow cytometry
[x]	[ ]	MRI-based neuroimaging

Antibodies

Antibodies used

Flow Cytometry
Rat anti-mouse CD31 PE-Cy7, BD Biosciences #561410 1:1000
Rat anti-mouse CD34 A647, BD Biosciences #560230
Rat anti-mouse CD26 PE, Biolegend #137803
Rat anti-mouse PDGFRa BV605, Biolegend #13916
Rat anti-mouse hematopetic cocktail PerCPCy5.5 BD Biosciences #51-9006964
Rat anti-mouse CD144 BV421 Biolegend #138013
Zombie-AQUA FVS kit Biolgend #423102

7'AAD viability staining Biolegend # 420404

Antibody used for IF
Rat anti-mouse CD31 BD Pharmingen #562939
Rabbit anti-SOX9 Merck Millipore #AB5535
Chicken anti-GFP Invitrogen #A10262
Rabbit anti-SLUG Abcam #AB27568
Rabbit anti-aSMA Abcam #AB7818
Rabbit anti-Cre Abcam #AB190117
Rabbit anti-activated Notch1 Abcam #AB8925
Rabbit anti-RBPJ Abcam #AB25949
Rabbit anti-ERG Abcam #AB92513

Secondary conjugated antibody
Goat anti-Rat IgG conjugated to AlexaFluo 568 Invitrogen A-11077
Goat anti-rabbit IgG conjugated to AlexaFluo 647 Invitrogen A32733

Validation

All antibodies used in this study is validated by manufacturer and all relevant citations are provided by the manufacturer's website. In addition, throughout the process of this work, we have validated the following antibody in western blot, ligand activation or the addition of inhibitors: anti-SOX9 (AB5535) and anti-activated Notch1 (AB8925)

Primary IF antibodies
Rat anti-mouse CD31 BD Pharmingen #550274
Baldwin HS, Shen HM, Yan HC, et al. Platelet endothelial cell adhesion molecule-1 (PECAM-1/CD31): alternatively spliced, functionally distinct isoforms expressed during mammalian cardiovascular development. Development. 1994;120(9):2539-2553.

Rabbit anti-SOX9 Merck Millipore #AB5535
Xue Y, Lian W, Zhi J, et al. HDAC5-mediated deacetylation and nuclear localisation of SOX9 is critical for tamoxifen resistance in breast cancer. Br J Cancer. 2019;121(12):1039-1049. doi:10.1038/s41416-019-0625-0

Chicken anti-GFP Invitrogen #A10262
Oh Y, Lai JS, Mills HJ, et al. A glucose-sensing neuron pair regulates insulin and glucagon in Drosophila. Nature. 2019;574 (7779):559-564. doi:10.1038/s41586-019-1675-4

Rabbit anti-SLUG Abcam #AB27568
Gou WF, Zhao Y, Lu H, et al. The role of RhoC in epithelial-to-mesenchymal transition of ovarian carcinoma cells. BMC Cancer. 2014;14:477. Published 2014 Jul 1. doi:10.1186/1471-2407-14-477

Rabbit anti-aSMA Abcam #AB7818
Zhou J, Wang XH, Zhao YX, et al. Cancer-Associated Fibroblasts Correlate with Tumor-Associated Macrophages Infiltration and Lymphatic Metastasis in Triple Negative Breast Cancer Patients. J Cancer. 2018;9(24):4635-4641. Published 2018 Nov 24. doi:10.7150/jca.28583

Rabbit anti-Cre Abcam #AB190177
Davidson LA, Callaway ES, Kim E, et al. Targeted Deletion of p53 in Lgr5-Expressing Intestinal Stem Cells Promotes Colon Tumorigenesis in a Preclinical Model of Colitis-Associated Cancer. Cancer Res. 2015;75(24):5392-5397. doi:10.1158/0008-5472.CAN-15-1706

Rabbit anti-activated Notch1 Abcam #AB8925
Fu, R., Lv, WC., Xu, Y. et al. Endothelial ZEB1 promotes angiogenesis-dependent bone formation and reverses osteoporosis. Nat Commun 11, 460 (2020). https://doi.org/10.1038/s41467-019-14076-3

Rabbit anti-RBPJ Abcam #AB25949
Mouillesseaux, K., Wiley, D., Saunders, L. et al. Notch regulates BMP responsiveness and lateral branching in vessel networks via SMAD6. Nat Commun 7, 13247 (2016). https://doi.org/10.1038/ncomms13247
Rabbit anti-ERG Abcam #AB92513
Tiwari, R., Manzar, N., Bhatia, V. et al. Androgen deprivation upregulates SPINK1 expression and potentiates cellular plasticity in prostate cancer. Nat Commun 11, 384 (2020). https://doi.org/10.1038/s41467-019-14184-0

Flow Cytometry antibodies
Rat anti-mouse CD31 PE-Cy7, BD Biosciences #561410 1:1000
Christofidou-Solomidou M, Nakada MT, Williams J, Muller WA, DeLisser HM. Neutrophil platelet endothelial cell adhesion molecule-1 participates in neutrophil recruitment at inflammatory sites and is down-regulated after leukocyte extravasation. J Immunol. 1997;158(10):4872-4878

Rat anti-mouse CD34 A647, BD Biosciences #560230
Zhang, Y., Roos, M., Himburg, H. et al. PTPσ inhibitors promote hematopoietic stem cell regeneration. Nat Commun 10, 3667 (2019). https://doi.org/10.1038/s41467-019-11490-5

Rat anti-mouse CD26 PE, Biolegend #137803
Sen A, Rothenberg ME, Mukherjee G, et al. Innate immune response to homologous rotavirus infection in the small intestinal villous epithelium at single-cell resolution. Proc Natl Acad Sci U S A. 2012;109(50):20667-20672. doi:10.1073/pnas.1212188109

Rat anti-mouse PDGFRa BV605, Biolegend #135916
Himburg HA, Termini CM, Schlussel L, et al. Distinct Bone Marrow Sources of Pleiotrophin Control Hematopoietic Stem Cell Maintenance and Regeneration. Cell Stem Cell. 2018;23(3):370-381.e5. doi:10.1016/j.stem.2018.07.003

Rat anti-mouse hematopetic cocktail PerCPCy5.5 BD Biosciences #51-9006964
Morrison SJ, Wandycz AM, Hemmati HD, Wright DE, Weissman IL. Identification of a lineage of multipotent hematopoietic progenitors. Development. 1997;124(10):1929-1939.

Rat anti-mouse CD144 BV421 Biolegend #138013
Corada M, Zanetta L, Orsenigo F, et al. A monoclonal antibody to vascular endothelial-cadherin inhibits tumor angiogenesis without side effects on endothelial permeability. Blood. 2002;100(3):905-911. doi:10.1182/blood.v100.3.905

Eukaryotic cell lines

Policy information about cell lines

Cell line source(s)	No cell lines were used in this study. ECFCs are primary cells isolated from the term placenta
Authentication	Primary ECFCs isolated were subjected to morphological, expression and functional tests based on protocol previously published doi: 10.5966/sctm.2013-0092
Mycoplasma contamination	Primary cells in cultured were tested negative for mycoplasma contamination
Commonly misidentified lines (See ICLAC register)	No commonly misidentified cell lines were used in this study

Animals and other organisms

Policy information about studies involving animals; ARRIVE guidelines recommended for reporting animal research

Laboratory animals	Both males and females (ages 8-14 weeks of age; genders housed separately) were used for this study. C57BL/6 mice (WT) were obtained from the Animal Resources Centre (Perth, Western Australia). For lineage tracing experiments Cdh5-CreERt2 were crossed with ROSAloxYFPlox, resulting in the double transgenic line Cdh5CreERt2/ROSAloxYFPlox. For endothelial specific KO of Rbpj, Sox9 and Ptch1, Rbpjfl/fl, Sox9fl/fl and Ptch1fl/fl mice were crossed with Cdh5CreERt2/ROSAloxYFPlox to generate the triple-transgenic knockout lines.
Wild animals	No wild animals were used in this study
Field-collected samples	No field collected samples were used in this study
Ethics oversight	All mice were treated in accordance with University of Queensland ethics approvals and guidelines for care of experimental animals. Animal ethics granted by University of Queensland Molecular Biosciences Animal Ethics Council under AE472/18 and AE473/18

Note that full information on the approval of the study protocol must also be provided in the manuscript.

Human research participants

Policy information about studies involving human research participants

Population characteristics	Healthy pregnant women with elected caesarean birth
Recruitment	Patients were recruited and informed written consent was obtained prior to their surgery.
Ethics oversight	The University of Queensland and the Royal Brisbane and Women’s Hospital under ethics: HREC/09/QRBW/14

Note that full information on the approval of the study protocol must also be provided in the manuscript.

Flow Cytometry

Plots

Confirm that:

- [x] The axis labels state the marker and fluorochrome used (e.g. CD4-FITC).
- [x] The axis scales are clearly visible. Include numbers along axes only for bottom left plot of group (a 'group' is an analysis of identical markers).
- [ ] All plots are contour plots with outliers or pseudocolor plots.
- [x] A numerical value for number of cells or percentage (with statistics) is provided.

Methodology

Sample preparation	To prepare a single-cell suspension for flow cytometry, mice were culled using CO2 asphyxiation. Aortas were immediately dissected, finely chopped using sterile surgical scissors, and incubated for enzymatic digestion under gentle agitation in 37oC Hanks' Balanced Salt Solution (BioWhittaker, MD, USA) supplemented with 150mg/mL DNAse-I (Sigma-Aldrich, MO, USA) and 1mg/mL of both collagenase I and dispase (Gibco, NY, USA). After 45 minutes of enzymatic digestion, the solution was mechanically dissociated through 70µm nylon mesh filters. Wounds were dissociated mechanically without enzymatic digestion. Excess erythrocytes were removed using a 30 second exposure to a hypertonic lysis solution (10mM NaHCO3, 150mM NH4Cl). The filtered single-cell suspensions were centrifuged at 500g after which the supernatant was removed. The cell pellet was resuspended into FACS buffer comprising of 1X PBS (Gibco, PA, UK), 2mM ethylenediaminetetraacetic acid (Sigma-Aldrich, MO, USA) and 0.5% bovine serum albumin (Sigma-Aldrich, MO, USA).
Instrument	Gallios flow cytometer, BD Fortessa X-20, BD FACSAria sorter
Software	Kaluza, BD FACSDiva, FlowJo
Cell population abundance	Lineage tracing reporter mice were used allowing the analysis of YFP+ endothelial populations. Cre-activated YFP+ cells were not significantly different between the knockout transgenic mouse lines.
Gating strategy	Cells were gated by FSC/SSC gates and then FSC/FSC-width to select single cells. Specific gating strategy is found in supplemental figure 3A

- [x] Tick this box to confirm that a figure exemplifying the gating strategy is provided in the Supplementary Information.
